# REPLY TO ARTICLE: CONSUMPTION OF ULTRA-PROCESSED FOODS BY CHILDREN UNDER 24 MONTHS OF AGE AND ASSOCIATED FACTORS

**DOI:** 10.1590/1984-0462/2020/38/2020139

**Published:** 2020-11-06

**Authors:** Karin de Almeida, Luana Lima da Silva, Eliane Mazzuco dos Santos

**Affiliations:** a Universidade do Sul de Santa Catarina, Tubarão, SC, Brazil.

Dear authors,

It is true that changes in dietary pattern have increased in the past decades, thus providing room for the growth of comorbidities related to weight gain, mainly among children. Therefore, the rise of ultra-processed foods, especially in the diet of children aged under 24 months of age, played an essential role for the changes in dietary pattern, and can be attributed to several factors, such as the return of the maternal figure to the work place, and the consequent early weaning.^1^

This study, carried out in the State of Minas Gerais, identified that 74.3% of the analyzed infants, mainly those aged more than 6 months, already consumed ultra-processed foods.^1^ On the other hand, another study in the same State showed that exclusive breast feeding until the age of 6 months occurred only among 24.9% of the participants, and that only 49% of the mothers participated in groups about breast feeding.^2^ Such data, found in different studies, agree that weaning is, in fact, early among Brazilian families.

A study, carried out to update maternal breast feeding indicators in Brazil in the last three decades, showed there was an increasing tendency until 2006; after that decade, the numbers became stable, which led to national warning, since recent studies reinforce the protection breast feeding provides against infectious diseases, besides leading to lower risk of chronic conditions.^3^

In this sense, the intake of human milk is associated to the reduced consumption of ultra-processed foods, thus showing the indispensability of actions to change the current breast feeding scenario, leading to benefits such as improved dietary habits and fewer child health problems.^4^

Therefore, the question is: how would it be possible to increase the number of breast-feeding months, and, consequently, reduce the introduction of ultra-processed foods?

The conclusion is that the article in question does not present the strategic actions that can be used to fight this problem. It is necessary to reassess the established public policies, proposing new strategies of intervention in Primary Health care, in order to provide more social engagement and support to breast feeding. Besides, it is important to train health professionals to advise and support the mothers, thus increasing the responsibility of the Family Health Strategy (ESF) to provide information and care to all of its users, especially the most vulnerable populations. Therefore, it is necessary to search for efficient tools to increase the breast-feeding period, in order to promote better quality of life to the children.
