# Supplementary material for: REPLY TO ARTICLE: CONSUMPTION OF ULTRA-PROCESSED FOODS BY CHILDREN UNDER 24 MONTHS OF AGE AND ASSOCIATED FACTORS
Source: Rev Paul Pediatr. 2020 Nov 6;38:e2020139. doi: 10.1590/1984-0462/2020/38/2020139 (PMC7649859; doi:10.1590/1984-0462/2020/38/2020139)
Supplement: Related texts

# Reply: LETTER-RESPONSE

**Authors:** Wanessa Casteluber Lopes, Lucinéia de Pinho, Antônio Prates Caldeira, Angelina do Carmo Lessa

**Affiliations:** a Universidade Federal dos Vales do Jequitinhonha e Mucuri, Diamantina, MG, Brazil.; b Universidade Estadual de Montes Claros, Montes Claros, MG, Brazil.

Dear editors,

The article in question did not aim at assessing or proposing intervention strategies, but instead, the objective was that described in the last paragraph of the introduction: “… to assess the intake of ultra-processed foods by children in the city of Montes Claros, aged under 24 months, and to identify its associated factors…”. It was based on a diagnostic and observational study. Therefore, the obtained results, the discussion and the final conclusions were based on a study of such a design.^1^

However, since the discussion of the article is also a place where the authors can issue their opinion on the findings,^2^ we point to an alternative described in the fifth paragraph of the discussion: “The association observed here, between the care taker and the early intake of ultra-processed foods, has to be considered from the perspective of programs addressed to the promotion of health diets for children…”. Therefore, the study provides evidence that can assist health professionals and administrators in the definition of policies and programs destined to this group.

In the conclusion of the article, we mentioned that “Therefore, this study can contribute with the orientation of health professionals in the counselling of families regarding the diet of children in the early years of life, emphasizing the proper introduction of complementary feeding, thus discouraging the introduction of ultra-processed foods”.

It is important to mention that diagnostic studies are carried out to scientifically support and contribute with intervention studies and research. And this study was relevant to point out the high intake of ultra-processed foods, which are introduced early in children’s diet.

We invite academics, professors, researchers and health professionals to invest more in interventions and tools, and to publish analyses on the subject, so that it is possible to evaluate better strategies to increase the time of breast feeding, to improve the adequation of complementary feeding, and to discourage the use of ultra-processed foods in this age group.

Finally, we understand that the comment is, above all, a reflection that is added to the interest of the authors to raise awareness for the theme. Considering that most readers are health professionals, many of whom in decision-making positions in health policies and programs, we expect that our study can assist them in this sense.

## References

[1] Rothman KJ, Greenland S, Lash TL (2008). Modern Epidemiology.

[2] Pereira MG (2013). A seção de discussão de um artigo científico. Epidemiol Serv Saúde.
